# β-caryophyllene emitted from a transgenic *Arabidopsis* or chemical dispenser repels *Diaphorina citri*, vector of *Candidatus* Liberibacters

**DOI:** 10.1038/s41598-017-06119-w

**Published:** 2017-07-17

**Authors:** Berta Alquézar, Haroldo Xavier Linhares Volpe, Rodrigo Facchini Magnani, Marcelo Pedreira de Miranda, Mateus Almeida Santos, Nelson Arno Wulff, Jose Mauricio Simões Bento, José Roberto Postali Parra, Harro Bouwmeester, Leandro Peña

**Affiliations:** 1 Laboratório de Biotecnologia Vegetal, Pesquisa & Desenvolvimento, Fundo de Defesa da Citricultura (Fundecitrus), Vila Melhado, 14807-040 Araraquara, São Paulo Brazil; 2 Instituto de Biología Molecular y Celular de Plantas (IBMCP), Consejo Superior de Investigaciones Científicas (CSIC)-Universidad Politécnica de Valencia (UPV), 46022 Valencia, Spain; 3 Chemistry Department, Universidade Federal de São Carlos (UFSCar), São Carlos, São Paulo Brazil; 4 Departamento de Entomologia e Acarologia, Escola Superior de Agricultura Luiz de Queiroz, Universidade de São Paulo, Piracicaba, São Paulo Brazil; 5 Swammerdam Institute for Life Sciences, University of Amsterdam, Science Park 904, 1098 XH Amsterdam, Netherlands

## Abstract

Production of citrus, the main fruit tree crop worldwide, is severely threatened by Huanglongbing (HLB), for which as yet a cure is not available. Spread of this bacterial disease in America and Asia is intimately connected with dispersal and feeding of the insect vector *Diaphorina citri*, oligophagous on rutaceous host plants. Effective control of this psyllid is an important component in successful HLB management programs. Volatiles released from the non-host guava have been shown to be repellent to the psyllid and to inhibit its response to citrus odour. By analysing VOC emission from guava we identified one volatile compound, (*E*)-β-caryophyllene, which at certain doses exerts a repellent effect on *D*. *citri*. Non-host plant rejection mediated by (*E*)-β-caryophyllene is demonstrated here by using *Arabidopsis* over-expression and knock-out lines. For the first time, results indicate that genetically engineered *Arabidopsis* plants with modified emission of VOCs can alter the behaviour of *D*. *citri*. This study shows that transgenic plants with an inherent ability to release (*E*)-β-caryophyllene can potentially be used in new protection strategies of citrus trees against HLB.

## Introduction

Huanglongbing (HLB), or ‘yellow shoot disease’ in a Chinese dialect, originated in South-eastern Asia from where it expanded during the past centuries, to become the most devastating citrus disease worldwide. It is caused by three species of phloem-limited *Candidatus* Liberibacter α-proteobacteria, which are transmitted from infected to healthy trees by grafting or by psyllid insect vectors, mainly the Asian citrus psyllid *Diaphorina citri* Kuwayama (Hemiptera: Liviidae) in Asia and America^1, 2^. Affected trees can maintain a healthy appearance for months or even years before the first symptoms of HLB infection are manifested, hindering early disease diagnosis and thus fast control. Eventually, if no treatment is applied, trees die in 5–10 years^1, 2^. Meanwhile, branches dry out gradually, while fruit yield is severely reduced (30–100%) and fruits taste bitter, thus limiting their commercial value. Together this generates substantial economical loses in heavily affected areas. Consequently, HLB is seriously threatening citrus production, which is first ranked among fruit tree crops in the international trade based on value, with an annual global production of more than 135 million metric tons^3^. In Florida, which is the largest citrus producing area in the USA (3^rd^ producing country worldwide)^3^, since HLB was first detected in 2005, sweet orange acreage, yield and production have been reduced by more than 37, 30 and 39%, respectively, until 2014^3^. Between the 2006–2007 and 2013–2014 seasons, the cumulative total impact of HLB in Florida, without considering the fresh fruit market or grapefruit for processing, has been estimated to amount to $7.902 billion in industry output and 60101 fewer jobs^4^.

HLB host range includes all known *Citrus* genotypes and to date no resistant germplasm has been reported for the genus. Current HLB management strategies involve all aspects of an integrated pest and disease management program: eradication of all existing sources of HLB within an area, replanting with HLB-free material, and use of intensive insecticide programs against the psyllid vector. So far, despite all the efforts made in last years to find a cure for HLB, there is no effective strategy for its sustainable control. *Ca*. Liberibacter species are obligate parasites of plants and the psyllids, and attempts to culture these bacteria *in vitro* have failed, making them difficult to study. In addition, genome sequencing of bacterial species associated with HLB has not improved our understanding of their pathogenicity mechanisms. Thus, efforts aimed to control psyllid populations are essential to mitigate HLB infection and spread.

In 2006, Beattie *et al*.^5^ reported that citrus interplanted with guava (at 1:1 ratio) in Vietnam had lower psyllid infestation and, as a consequence, lower HLB incidence than citrus planted alone. A reduction of 33 to 52% in the number of psyllids settling on citrus leaves when guava foliage is also present has been reported in choice and no-choice assays, respectively^6, 7^. By olfactometric assays it was demonstrated that this effect was due to guava volatiles, which reduce attraction of the psyllid to citrus plants by repelling them or interfering with their ability to locate their host^7, 8^. In addition, oil extracts from leaves of different guava cultivars were repellent to *D*. *citri* ^6^. A ‘push’ strategy consisting of interplanting citrus and guava trees to deter the psyllids would be a durable strategy to limit HLB spreading. This kind of strategy, rarely used in western agriculture, is being successfully employed for example in East Africa, where maize yields were increased more than three times by controlling insect pests and parasitic weeds through a push-pull strategy consisting of interplanting maize with Napier grass (as pull) and molasses grass/forage legumes (as push)^9^. However, interplanting citrus with guava would reduce yield per area and increase grove management costs. Moreover, the guava protective effect decayed when citrus trees started to exceed the height of the guava trees^10^. A promising alternative would be to identify which emitted volatile compound/s is/are responsible for the repellent effect of guava, and to use it/them to develop effective and successful integrated pest management. However, up to date, research regarding guava volatile organic compounds (VOCs) has ignored emission, and only focussed on the content^6, 11–17^, precluding identification of VOCs involved in *D*. *citri* repellence. Our goal was hence to identify the volatile chemical/s involved in the guava effect and to test the possibility of increasing their production in transgenic plants for developing a psyllid-push control strategy.

## Results and Discussion

**Figure 1:**
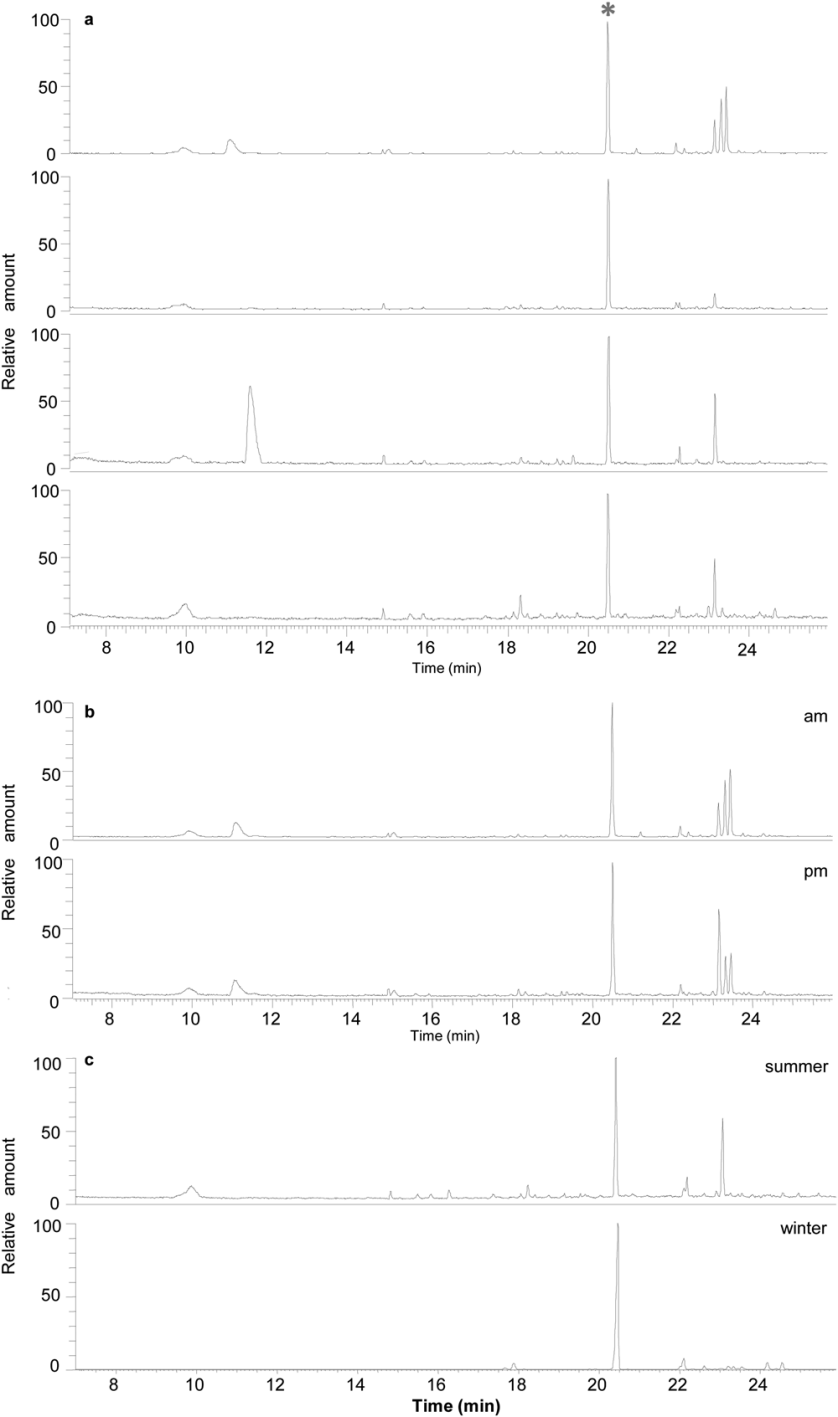
Representative gas chromatographic mass spectrometry separation of volatiles emitted from guava leaves under different conditions: (**a**) VOC emission from leaves in different developmental stages (from top to bottom: flush; young; mature; old); (**b**) VOC emission from flushes at different time points; (**c**) VOC emission from old leaves in different seasons. *, β-caryophyllene.

**Figure 2:**
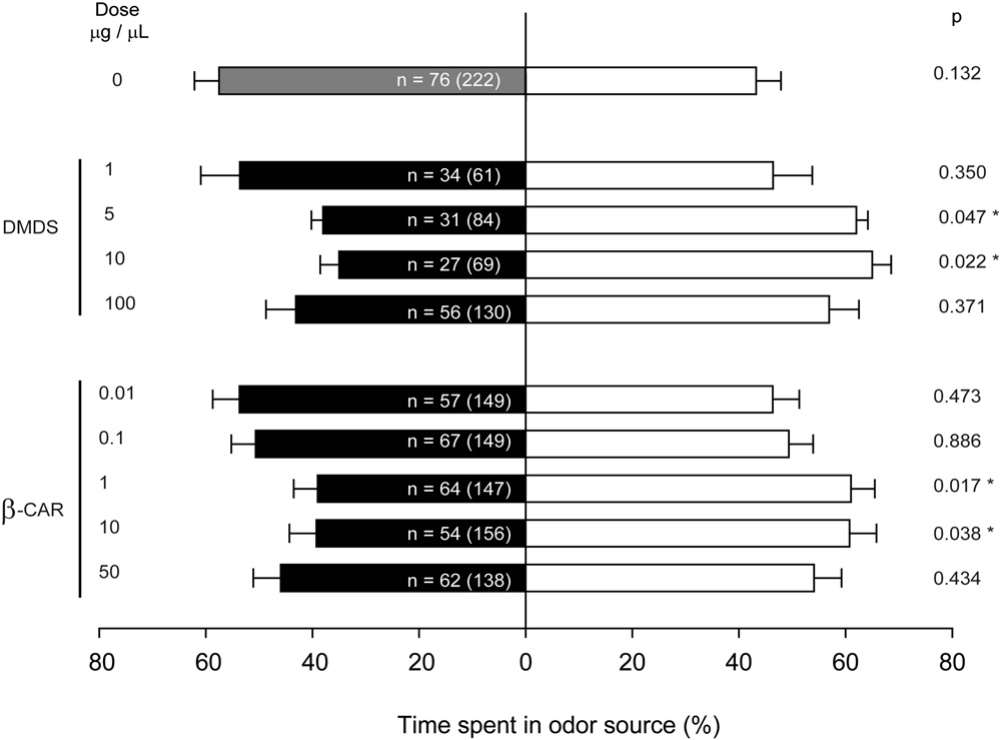
Responses of *D*. *citri*, tested in a 4-arm-olfactometer, to different concentrations of two synthetic volatiles, dimethyl disulphide (DMDS) and β-caryophyllene (β-car) dissolved in hexane. Bars represent the overall percentage of time spent in each odor source, comparing air (grey bar) or a synthetic volatile (black bar) versus hexane (white bar). Error bars represent SE (n = 10 independent tests). The number of responding psyllids is indicated in the black bars and the total number of insects tested is given between parentheses for each comparison. Time spent in odor sources were analysed by paired *t*-test. Asterisks indicate significant differences between odor sources (*P < 0.05).

To identify the volatile/s mediating guava-repellent effect, we analysed which chemicals are emitted from intact guava leaves by headspace solid phase microextraction, which provides a more representative overview of the volatile profile emitted by living plants than traditional methods of solvent extraction or steam distillation^18^. As the guava effect is present all year round^19^ but plant emission profiles may vary depending on leaf developmental stage, environmental conditions and even the hour of the day^18^, VOC analyses were performed under a range of conditions (Fig. 1). In all samples, sesquiterpenes were the predominant volatiles and (*E*)-β-caryophyllene (from now on referred to as β-caryophyllene) was the major VOC, constituting between 20.61 ± 3.80% and 58.92 ± 7.44% of total VOCs. Coincidently, one of the most abundant VOCs present in guava oil extracts is also β-caryophyllene, independently of the cultivar and the location^6, 11–17^. On the other hand, citrus leaves release mainly (more than 80%) monoterpenes, while β-caryophyllene is barely emitted^20, 21^ (Suppl. Fig. 1). Thus, its abundance and continuous emission from guava leaves and its absence in citrus leaf emission profiles suggest that β-caryophyllene may be responsible for the repellence of guava leaves to the psyllid. To test this, *D*. *citri* response to β-caryophyllene was evaluated in a 4-arm olfactometer (Fig. 2). Dimethyl disulfide (DMDS), which has been reported as a potent *D*. *citri* repellent^8, 19^ was used as control. When tested with clean air, the psyllid responded equally to each of the four arms, indicating no positional bias in the bioassay (Suppl. Fig. 2). Overall, the mean percentage of psyllids that made a choice during the various olfactometer tests was 42.30 ± 1.79 (%, mean ± SE), ranging from 34.23 to 55.74%. This range matches with studies of others (24–68%), in which the response of *D*. *citri* was also evaluated in a 4-arm olfactometer^8^.

Similarly to guava leaves, which had no repellent effect at low doses (when just a few leaves were assayed, i.e. below 2.5 g of fresh weight)^7^, 0.01 to 0.10 μg of β-caryophyllene per μL of hexane did not elicit a response in the psyllid (Fig. 2). β-caryophyllene had a repellent effect from a dose of 1 μg/μL (Fig. 2), about 5-fold less than the minimum dose of DMDS required to repel the psyllid according to both our results (500 μg, Fig. 2) and those of other authors^8^, who showed that 430 μg was the lowest dose of DMDS affecting attraction of *D*. *citri* to citrus volatiles. Our result fits well with the high response of psyllid olfactory receptor neurons (ORNs) to β-caryophyllene, which could be detected by the psyllid at the lowest concentration among more than 100 volatiles tested^22^. The repellent effect of β-caryophyllene was maintained at least up to a dose ten-time higher, but it was lost when 50 μg/μL (5 mg) of compound was evaluated. Also in a T-maze olfactometer, 5 mg of β-caryophyllene elicited no response in *D*. *citri* ^23^. The loss of the repellent effect of both β-caryophyllene and DMDS at higher doses is not well understood and needs more investigation, but could perhaps be related to antennal receptor saturation or desensitization to odour, as has been proposed for the attraction response of *Drosophila* larvae^24^. Recently, it has also been proposed that high doses of the *D*. *citri* attractant methyl salicylate may disrupt their sensory capability^25^.

**Figure 3:**
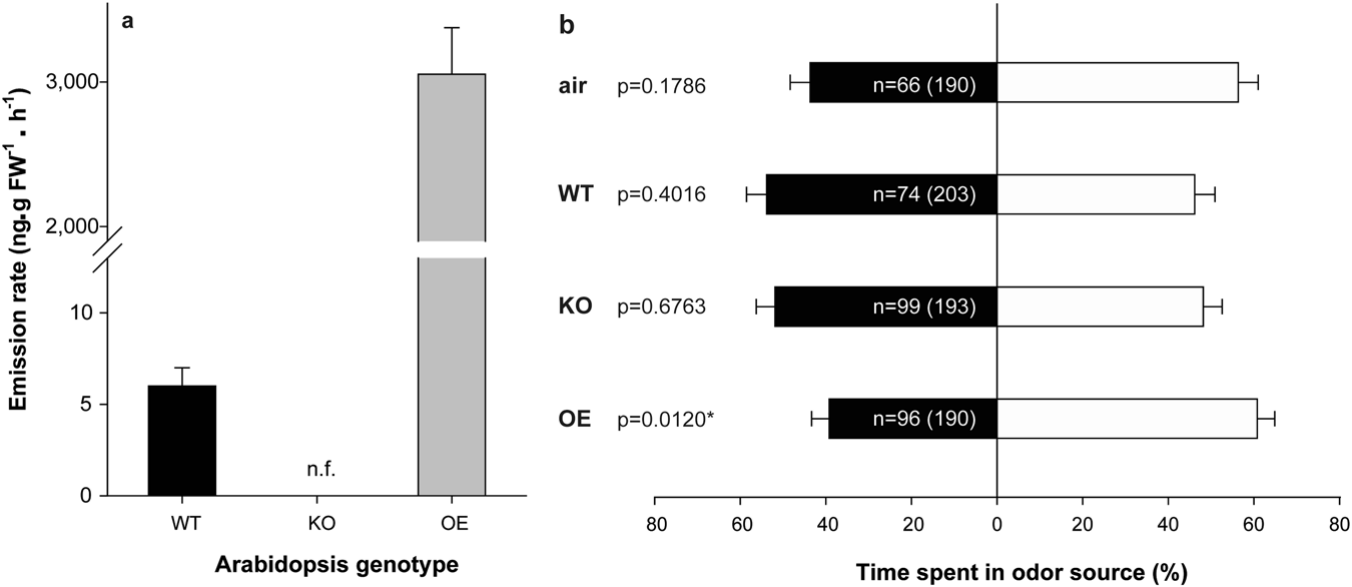
(**a**) Quantitative measurement of β-caryophyllene emitted by flowering plants of different *Arabidopsis thaliana* (Col-0 background) accessions: wild type (WT), β-caryophyllene knock-out mutant of At5g23960 (KO) and transgenic line β-caryophyllene synthase-overexpressing At5g23960 (OE). n.f., not found. (**b**) Responses of *D*. *citri*, tested in a 4-arm-olfactometer, to volatiles of two sets of odour sources. WT, KO and OE (see above) *Arabidopsis thaliana* (black bars) lines were tested against pots with substrate (withe bars). Bars represent the overall percentages of time spent in each of the two odour sources. Error bars represent SE (n = 10 independent test). The number of responding psyllids is indicated in the black bars and the total number of insects tested is given between parentheses for each comparison. Time spent in odor sources were analysed by paired *t*-test. Asterisks indicate significant differences between odour sources (*P < 0.05).

Modification of volatile blends by genetic engineering has been shown to modify the behaviour of insects in relation to plants. For example, *Arabidopsis* plants engineered for producing (3*S*)-(*E*)-nerolidol and (*E*)-4,8-dimethyl-1,3,7-nonatriene or a blend of sesquiterpenes, became attractive to carnivorous predatory mites^26^ or to parasitic wasps^27^, respectively, while linalool-producing *Arabidopsis* plants became repellent to the aphid *Myzus persicae* ^28^. We wondered then if induced emission of β-caryophyllene could make the non-host plant *Arabidopsis thaliana* repellent to *D*. *citri*. To that end we generated a β-caryophyllene synthase (At5g23960) overexpression line emitting much more β-caryophyllene (about three orders of magnitude higher) than Col-0 wild type (WT) plants^29^ (Fig. 3a, Suppl. Fig. 3). We also used an At5g23960 knock out (KO) line, which does not produce β-caryophyllene^30^. Response of *D*. *citri* to plants from all three lines (OE, KO and WT), which showed comparable numbers of open flowers, closed flowers and siliques (Table S1), were tested in four-arm olfactometer assays. Psyllids exposed to the WT and KO line did not show any response, indicating that *Arabidopsis* itself with or without β-caryophyllene had no repellent effect on *D*. *citri* (Fig. 3b). Conversely, when the OE line was assayed, psyllids spent significantly less time in the transgenic odour fields, thus confirming a repellent effect of β-caryophyllene on the insect. By comparing the mean time that psyllids spent in clear air fields when exposed to KO and OE lines, a repellence index of 24.68 ± 2.26% was obtained for OE. The WT line did not exhibit any repellent effect on the psyllids, despite the fact that their flowers emit a complex mixture of terpenes with β-caryophyllene as the dominant one^31^. However, as other self-pollinating plants, the rate of VOC emission from *Arabidopsis* Col-0 flowers is small^31^ (Fig. 3a), so this result is not surprising as β-caryophyllene and guava leaves were not repellent at low doses^7^ (Fig. 2). Interestingly, β-caryophyllene emission by the OE line in olfactometer assays was comparable to that of the standard compound when used at 1 μg/μl, 0.10 ± 0.01 and 0.16 ± 0.01 ng/ml, respectively.

In recent years, it has been shown that plant-produced VOCs play an essential ecological role mediating the interactions between plants and other organisms. For example, it is now well known that foliar VOCs are among the most important cues that facilitate insect herbivores to locate host plants at a distance^32^. These olfactory cues are mediated mainly by the perception of specific blends or ratios of ubiquitous plant VOCs, rather than by specific compounds^32, 33^. So much so that VOCs functioning as attractive signals in the context of the total host volatile blend can act as repellents when offered individually^34^. Therefore, before developing a pest control strategy based on the use of a repellent VOC, it is important to check the effect of the compound in the aromatic context in which its use is desired. Thus, we also evaluated whether the repellent effect of β-caryophyllene was maintained in a psyllid-attractive citrus bouquet consisting of sweet orange flushes with small developing leaves which are preferred by *D*. *citri* for feeding and oviposition^35^. As expected, when the citrus leaves were supplemented with the minimum repellent dose of β-caryophyllene (at 1 μg/μL) they became less attractive to the psyllids, with a repellence index of 22.34 ± 2.36% (data not shown), illustrating the potential use of β-caryophyllene to make citrus hosts less attractive to *D*. *citri*.

**Figure 4:**
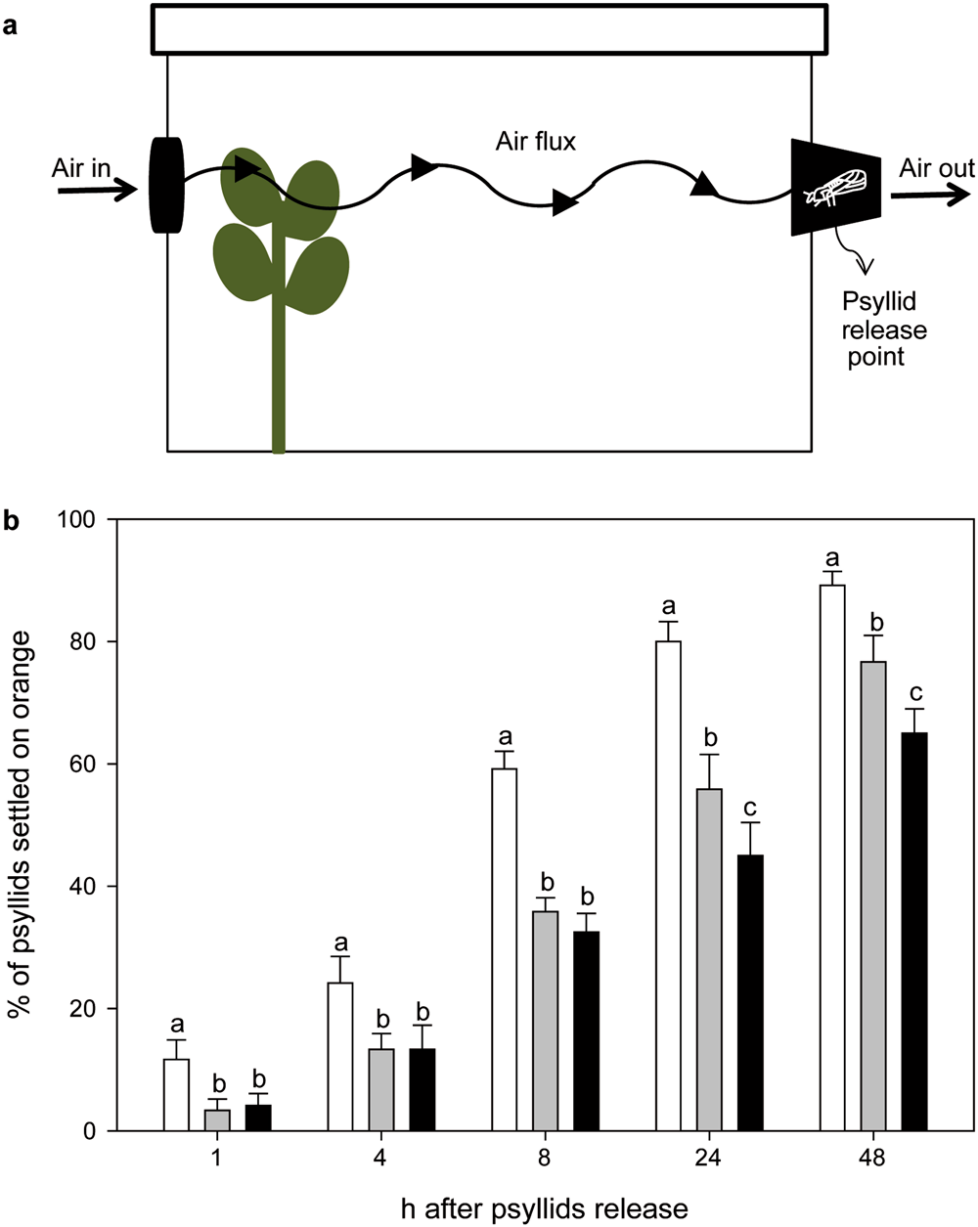
(**a**) Graphic representation of the device developed to evaluate in no-choice tests the effect of plant volatiles on *D*. *citri*. (**b**) Percentage of *D*. *citri* psyllids settled 1, 4, 8, 24 and 48 h after their release on sweet orange flushes when presented alone (white bars), caged with *Arabidopsis* At5g23960 KO line (grey bars) or caged with *Arabidopsis* At5g23960 OE line (black bars). For a given time, the bars with different letters indicate significant differences between the treatments tested (*P < 0.1). Error bars represent SE (n = 12 independent tests, 10 responsive psyllids evaluated in each test).

**Figure 5:**
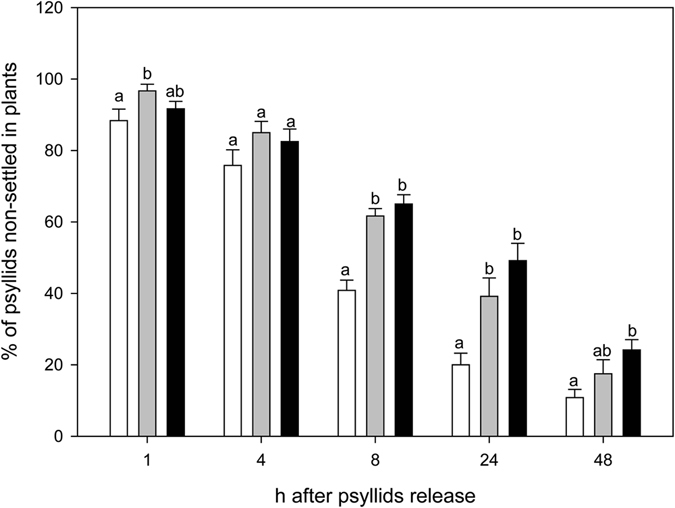
Percentage of *D*. *citri* psyllids non-settled in any plant (neither orange nor *Arabidopsis*) 1, 4, 8, 24 and 48 h after their release when orange flushes were presented alone (white bars), caged with *Arabidopsis* At5g23960 KO line (grey bars) or caged with *Arabidopsis* At5g23960 OE line (black bars). For a given time, the bars with different letters indicate significant differences between the treatments tested (*P < 0.1). Error bars represent SE (n = 12 independent tests, 10 responsive psyllids evaluated in each test).

Besides olfaction, *D*. *citri* also relies on visual cues for finding their host^36^. Moreover, in olfactory tests to assess attractiveness, a strong response is only obtained when olfactory cues are presented together with visual stimuli^37^. Consequently, β-caryophyllene influence on *D*. *citri* ability to find host plants was evaluated in behavioral assays with flushing sweet orange plants, combined with or without *Arabidopsis* lines emitting (OE) or not (KO) β-caryophyllene (Fig. 4; Suppl. Fig. 4). In each treatment, the number of psyllids settled on sweet orange leaves was counted over a period of 48 h. More than half (59.17 ± 2.88%) and almost all (80.00 ± 3.26, 89.17 ± 2.29%) of the released insects were able to locate the sweet orange plant when presented alone at 8, and 24–48 h after their release, respectively. At all the times analysed, when sweet orange plants were caged with *Arabidopsis*, the number of psyllids settled on the orange leaves diminished, between 1.37 and 3.50 fold, regardless of whether the *Arabidopsis* line emitted β-caryophyllene or not, indicating that non-host plants presence interfered with the ability of *D*. *citri* for localising their host. These results coincided with that of non-choice studies in which it was shown that the presence of guava, but also cotton, reduced the speed at which psyllids find and settle on the citrus host^38^. At all but one time of inspection, psyllids were found on non-host *Arabidopsis* plants (Suppl. Fig. 5), up to about 8.33 ± 0.01% at 48 h. Settling on non-host, such as guava, cotton, tomato and azalea (*Rhododendron simsii*) plants has also been reported before^38, 39^. On the other hand, 24 h after release of the psyllids, differences in the number of insects settled on sweet orange plants started to become visible between OE and KO treatments (Fig. 4). In those cages in which OE was used, the number of insects settled on sweet orange was reduced by 19.40 and 15.21% in comparison with KO, at 24 and 48 h, respectively. It is also interesting to note that the number of psyllids that were not able to find the citrus host was slightly increased in those trials in which the *Arabidopsis* β-caryophyllene overproducing line was used (Fig. 5). As these effects were attributable to the emission of β-caryophyllene by the OE line, it can be concluded that this compound actively interferes in the location of the host by the HLB vector. If compared to sweet orange plants presented alone, the reduction of *D*. *citri* settling on citrus leaves when the β-caryophyllene overproducing line was co-presented was 43.75 and 27.10% after 24 and 48 h, respectively (Fig. 4). These values are very close to the reductions in the number of psyllids found on citrus plants when grapefruit leaves were presented together with guava leaves as compared to just grapefruit leaves alone, about 50 and 30% after 1 and 2 days^38^.

In conclusion, we have identified a volatile compound from guava, namely β-caryophyllene, which at certain doses exerts a repellent effect on *D*. *citri* (Fig. 2). Other guava volatiles may contribute to its repellence^8, 40^. β-caryophyllene-induced repellence was evident in doses lower than that required for DMDS-induced repellence, and, in addition, the former does not present an unpleasant odour, so it could be used in the field through slow release chemical dispensers as was proposed for DMDS^8, 19^. However, the volatility, instability and production costs of β-caryophyllene may be problematic for use in agriculture. This could be solved by expressing the genes for their biosynthesis in plants of interest. The success of this strategy has already been demonstrated in transgenic tobacco lines with increased emission of patchoulol, linalool and isoprene that became less attractive to herbivory/oviposition than their wild type counterparts^41–43^. Also, transgenic tobacco, corn and wheat plants capable of producing β-farnesene, the main component of the alarm pheromone for many aphids, were unattractive/repellent for these insects^44–46^. In this work, we have demonstrated that the emission of β-caryophyllene at levels above a certain concentration has the ability of turning a neutral non-host plant to *D*. *citri* into a repellent one (Fig. 3) and that the effect that this compound exerts on the psyllid is maintained even if presented in a background of citrus volatiles (Figs 4 and 5). Although the olfactometer tests with commercial β-caryophyllene indicate that the compound is repellent to the psyllid, the cage assays carried out in combinations of transgenic *Arabidopsis* with sweet orange plants suggest that the emission of β-caryophyllene by the OE line confuses the insects that are therefore not able to detect the volatile mixture of their citrus host. Foreseeably, transgenic citrus plants overproducing β-caryophyllene would become less attractive or even repellent to the vector of HLB, thus limiting spreading the latter. These GM-citrus cultivars would themselves constitute a ‘push’ crop able to drive the psyllid out of the field, thereby avoiding the need of spending resources to manage a repellent intercrop. In the case of large monoculture fields, as those commonly found in Brazil and USA (2^nd^ and 3^rd^ world citrus producers), use of these transgenic citrus cultivars would change the habitat cues used by insects to detect the host over long distances^47^. Moreover, the ‘push’ effect may be supplemented by the use, as cover crop, of other psyllid-repellent plants, as *Eupatorium catarium*, *Lippia graveolens* and *Allium spp*.^48–50^. Alternatively, the cover crop may be a transgenic plant overproducing β-caryophyllene.

It has been reported that the efficiency of push and pull behaviour controlling elements is often not only additive but synergistic^51^, so the push strategy proposed here could be combined with a pull strategy to attract the psyllids. Orange jasmine (*Murraya paniculata* (L.) Jack) is a *D*. *citri* preferred host and its potential use as a trap for *D*. *citri* has been already indicated^52^. So, as *D*. *citri* populations are much more prevalent on crop borders^53^, planting orange jasmine on the outer edges of the groves would prevent psyllid movement into the crop. Intensive insecticide treatments on orange jasmine trap could greatly reduce *D*. *citri* population, which would be concentrated in these plants, especially if combined with β-caryophyllene-emitting citrus cultivars as push. Furthermore, the use of GM non-attractant/repellent β-caryophyllene over-emitting citrus plants for psyllid control may reduce the amount of insecticide treatments currently used to mitigate HLB expansion, which incurs huge economic and environmental costs^54^.

## Methods

### Plant and insect material

Guava (*Psidium guajava*) seeds obtained from local growers from Vietnam were grown in greenhouses for at least two years before performing analyses. Other sources of guava plants were commercial nurseries of Sao Paulo (Brazil). Leaves from mature plants of *Citrus sinensis*, *Citrus clementina* and *Citrus aurantifolia* were obtained from The Citrus Germplasm Bank at the Instituto Valenciano de Investigaciones Agrarias (Moncada, Valencia, Spain) and Fundecitrus (Araraquara, Sao Paulo, Brazil).

Seeds of *A*. *thaliana* (L.) Heynh. ecotype Col-0, At5g23960 (TPS21, Atβ-caryophyllene synthase) T-DNA insertion line Salk_136913C (N660201)^55^ obtained from the ABRC stock centre, and AtTPS21 overexpressing line^29^ were grown to the flowering state on soil in a controlled climate-chamber (22 °C, 55% RH) for up to 7 weeks under a L10:D14 photoperiod (3000 lux).

For olfactometer bioassays, 7–15 day-old mated *D*. *citri* females were drawn from a continuously reared culture at Fundecitrus (Araraquara, Sao Paulo, Brazil). This culture is maintained on *Murraya paniculata* (L.) Jack seedlings at 25 ± 2 °C, 65 ± 10% RH and L14:D10 photoperiod (3000 lux) and it is composed of *C*Las free psyllids.

### Volatile emission sampling and analysis by GC-MS

Static headspace sampling with a solid phase microextraction (SPME) device was performed to get a realistic picture of volatiles emitted by guava and citrus leaves. Analyses were conducted using leaves at different developmental stages (1) flush, young flushes of 3–5 cm length; (2) young, young leaves of 7–10 cm length; (3) mature, fully expanded leaves of 9–12 cm length and dark green colour and (4) old, full mature coriaceous leaves), collected at different hours of the day (9.00 a.m. and 18.00 p.m.) and in different seasons (summer and winter). Leaf samples were enclosed in 50 mL screw-cap Pyrex tubes carrying a septum on the top and containing 1 mL of milli-Q water for avoiding leaf hydric stress. SPME fiber (100 μm poly(dimethyl) siloxane, Supelco, Bellefonte, PA) was exposed, at a controlled temperature of 22 °C, between 1 and 4 h. Immediately afterwards, the fiber was transferred to the Gas Chromathograph (GC) injector (220 °C) and thermal desorption (TD) was applied for 4 min. GC-MS (mass spectrometer) analysis was carried out as described before^56^ using a Thermo Trace GC Ultra coupled to a Thermo DSQ mass spectrometer equipped with a HP-INNOWax (Agilent J&C Columns) column (30 m × 0.25 mm i.d. × 0.25 μm film).

Volatiles emitted by the various odour sources in the olfactometer were trapped during 15 min, at the same conditions used for behavioural tests, in TD tubes (0.635 × 8.89 cm, Supelco) filled with 200 mg Tenax^®^-TA, 35–60 mesh (Sigma-Aldrich, Bellefonte, PA, USA) and directly connected to the PTFE tubes that were carrying the odour from the chambers. A vacuum pump was directly connected to the thermal desorption tubes onto the outlet for sucking the air (0.4 litre per minute) out of the chambers. Collected volatiles were released thermally from the tubes in a TD-20 thermal desorption unit (Shimadzu, Japan) at 250 °C for 5 min under a helium flow of 20 ml min^−1^ while re-collecting the volatiles onto a secondary cold trap tube (−20 °C) packed with Tenax^®^ (35–60 mesh). Once the desorption process was completed, the cold trap was heated at 40 °C s^−1^ to 280 °C and was kept for 5 min at 280 °C, while the volatiles were released in a split mode (ratio of 1:10) to a dimethyl polysiloxane capillary column (30 m × 0.25 mm i.d. × 0.25 μm film, Restek, Bellefonte, PA). GC was coupled to a MS GCMS-QP2010 Plus (Shimadzu, Japan). The GC temperature program consisted of start temperature at 40 °C held for 1 minute, followed by a temperature ramp of 7 °C min^−1^ to 250 °C, and then held for 5 minutes. The detector interface and the ion source temperature were 250 °C, and the carrier gas was He (1.01 mL min^−1^, split ratio 1:10). Mass spectra were recorded at 70 eV and all analyses were done in the total ion chromatogram (TIC) mode with the mass scan range from *m/z* 40 to 500.

Compounds were tentatively identified by matching of the acquired mass spectra with those stored in reference libraries (NIST, MAINLIB, REPLIB, accessed over 2016). Identity of β-caryophyllene was confirmed by injection of authentic standard (Cat. N° 75541, Sigma Aldrich, Steinheim, Germany, ≥98.5% pure). Absolute quantifications of β-caryophyllene emission of samples used in *D*. *citri* olfactometer assays were done by using a calibration curve constructed with an authentic standard (Sigma-Aldrich).

### *D*. *citri* olfactometry assays

The preferences of *D*. *citri* toward volatiles were investigated using a 4-arm olfactometer^57^. Individual constant charcoal-filtered humidified air flows (0.4 L min^−1^) were connected to each odour source (plant, clean air or chemical compounds), and all air flows (0.4 L min^−1^ each one) converged through individual 0.635 cm-diameter polytetrafluoroethylene (PTFE) tubes (Sigma-Aldrich, Bellefonte, PA, USA) to a central acrylic piece (30 cm × 30 cm × 2.5 cm), where the psyllids were released. An olfactometer test consisted in the release of a single female and the observation of its behaviour during 10 min, recording the time spent in each odour field. Psyllids that did not perform a choice after 5 min were recorded as “no response” in the analysis of results. For each experiment the time spent in each odour source for each responding female was considered as a replicate and a minimum of 10 replicates were performed each day. A dataset from ten independent days (from 10:00 am to 4:00 pm) were considered in the analyses. All experiments were conducted at 25 ± 1 °C, 65 ± 5% RH and constant light (2300 lux).

For each treatment studied, two of the four possible arms received volatiles of interest, while the other two remained as controls (hexane or pots without plants if chemicals or plants were studied, respectively). Time spent in each odour source (control or treatment) for each insect was expressed as percentage. β-caryophyllene and dimethyl disulfide (≥98.5% pure each) were obtained from Sigma-Aldrich (Steinheim, Germany) and released in a 2 mL glass vial. 100 μl of solution in hexane were used for each observation. The chemical dispenser was changed after each tested insect (around 15 min interval) and glassware was cleaned daily. When *A*. *thaliana* lines were evaluated, the same plants were employed during the whole day of experiment. As inflorescences are the *Arabidopsis* organs emitting the highest amounts of volatiles^31^, flowering plants were used in order to maximize the emission of VOCs.

Repellence index (RI), reflecting the percentage of repellence of one treatment as compared with another, was calculated as RI = [(C − T)/C]*100^58^, being C and T the mean time spent in the clean air odour fields in control and treatment olfactometer assays, respectively. Orange flushes (terminal stems with buds and young leaves to 7 cm long) and *Arabidopsis* KO line versus clean air were considered control assays (C). T corresponds to β-caryophyllene tests, in which orange flushes supplemented with 1 μg/μL of β-caryophyllene or *Arabidopsis* OE line versus clean air were assayed.

### *D*. *citri* behavioural responses to olfactory and visual cues

A system was developed to evaluate the effect promoted by plant volatiles associated with visual cues (Fig. 4 and Suppl. Fig. 4). For this, two holes were made on two opposite sides of a transparent plastic box with lid (57 × 40 × 35 cm). In one side, a central processing unit (CPU) fan cooler connected to a potentiometer were coupled on a hole covered with voil tissue (7.5 cm in diameter). On the opposite side of the box, a black plastic cup was fixed on another hole (same size as used to connect the fan) by sticking the cup rim on the perimeter of the hole. The bottom of the cup was replaced by a voil tissue. A small hole was made in a side of the cup to release insects into the device and covered with a piece of sponge. A third hole (same diameter as used for the other two) was made on the bottom of the plastic box allowing the introduction of a *C*. *sinensis* var. Pera plant grafted on *C*. *limonia* containing three flushes (10–18 cm each). In this way, the fan allowed that introduced air can pass through the internal part of the box carrying on the plant volatiles to the insect direction and leave the device by the bottom of the cup. The air speed was set up to 0.55 m.s^−1^ and the correct settlement of the air flow was confirmed by a smoke test (Suppl. Video 1). Three treatments were tested. (A) one citrus plant; (B) one citrus plant plus six glass pots containing five *Arabidopsis* At5g23960 KO plants each one and (C) one citrus plant plus six glass pots with *Arabidopsis* At5g23960 OE plants. The *Arabidopsis* plants were placed three in each side of the citrus plant, allowing that the insect could have visual contact with the former (Suppl. Fig. 4, located in the central region of the box). Ten 7–15 days-old mated females were released and 12 replications per treatment were performed (each box was considered a replication). The number of settled insects on citrus plants was assessed 1, 4, 8, 24 and 48 h after insect’s release. The experiments were performed under same room conditions (temperature, photoperiod and relative humidity) described for olfactometer tests.

### Statistical analyses

Variance homogeneity and residual normality of data generated from 4-arm olfactometer tests were verified by the Bartlett^59^ and the Shapiro-Wilk^60^ tests, respectively. Since the data met the assumption of the normal model, they were submitted to a variance analysis (ANOVA) and the means were compared by paired t-test (p < 0.05).

In behavior assays, the number of psyllids settled on citrus plants was recorded at different times. As the data did not conform to simple variance assumptions, they were subjected to logistic regression and Chi square (χ^2^) analysis tests (p < 0.10) were used to determine whether the presence of different *Arabidopsis* lines was related to the proportion of psyllids settled on citrus flushes.

Analysis were performed using the statistical software “R” version 3.2.3^61^.

## Electronic supplementary material

Supplementary Video 1

Supplementary Information
