# Supplementary material for: β-caryophyllene emitted from a transgenic Arabidopsis or chemical dispenser repels Diaphorina citri, vector of Candidatus Liberibacters
Source: Sci Rep. 2017 Jul 17;7:5639. doi: 10.1038/s41598-017-06119-w (PMC5514130; doi:10.1038/s41598-017-06119-w)
Supplement: Supplementary file 2 — Supplementary Information [file 41598_2017_6119_MOESM2_ESM.pdf]

TITTLE: β-caryophyllene emitted from transgenic *Arabidopsis* or chemical dispenser repels *Diaphorina citri*, vector of *Candidatus* Liberibacters

AUTHORS

Berta Alquézar[1,2], Haroldo Xavier Linhares Volpe[1], Rodrigo Facchini Magnani[1,3], Marcelo Pedreira de Miranda[1], Mateus Almeida Santos[1], Nelson Arno Wulff[1], Jose Mauricio Simões Bento[4], José Roberto Postali Parra[4], Harro Bouwmeester[5], Leandro Peña[1,2,*]

[1]Laboratório de Biotecnologia Vegetal. Pesquisa & Desenvolvimento. Fundo de Defesa da Citricultura (Fundecitrus). Vila Melhado. 14807-040 Araraquara, São Paulo, Brasil.

[2]Instituto de Biología Molecular y Celular de Plantas (IBMCP). Consejo Superior de Investigaciones Científicas (CSIC)-Universidad Politécnica de Valencia (UPV). 46022 Valencia, España.

[3]Chemistry Department, Universidade Federal de São Carlos (UFSCar), São Carlos, São Paulo, Brazil.

[4]Departamento de Entomologia e Acarologia. Escola Superior de Agricultura Luiz de Queiroz. Universidade de São Paulo. Piracicaba, São Paulo, Brasil.

[5]Swammerdam Institute for Life Sciences. University of Amsterdam. Science Park 904. 1098 XH Amsterdam, Netherlands.

*Correspondence to lpenya@fundecitrus.com.br

Suppl. Fig. 1

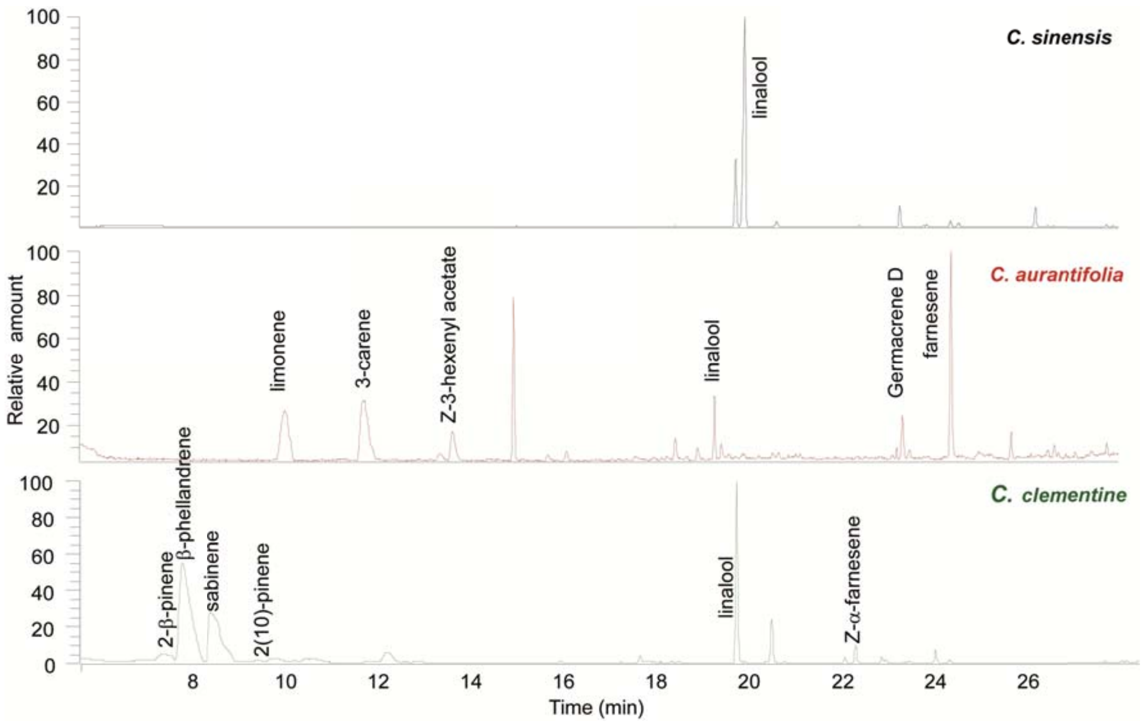

Representative gas chromatographic mass spectrometry separation of volatiles emitted from adult leaves of different *Citrus* species. Most representative VOCs are detailed.

Suppl. Fig. 2

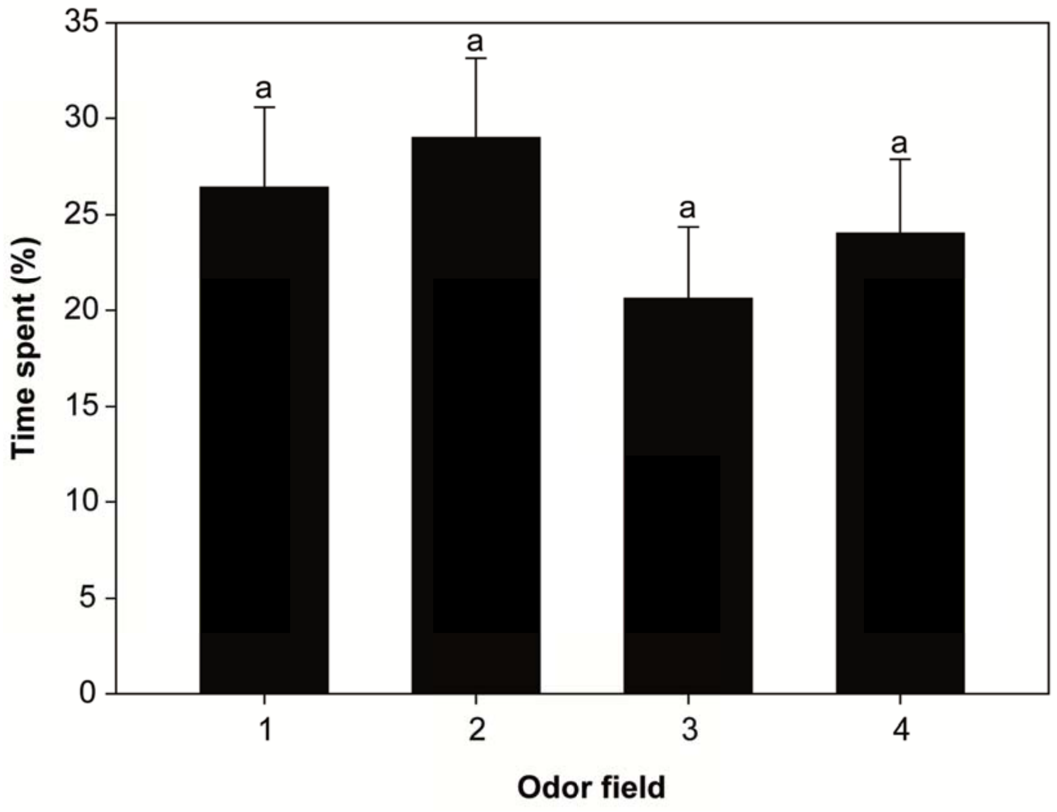

Time spent by *D. citri* in each of the four olfactometer arms when clean air was assayed in all them. Assays were performed in ten different days with at least 17 different insects per day and in total the response of 210 individuals was recorded. Mean response was 36.67 ± 3.08 %. Bars with same letter indicate that there is no significant difference between arms ($p > 0.05$).

Suppl. Fig 3

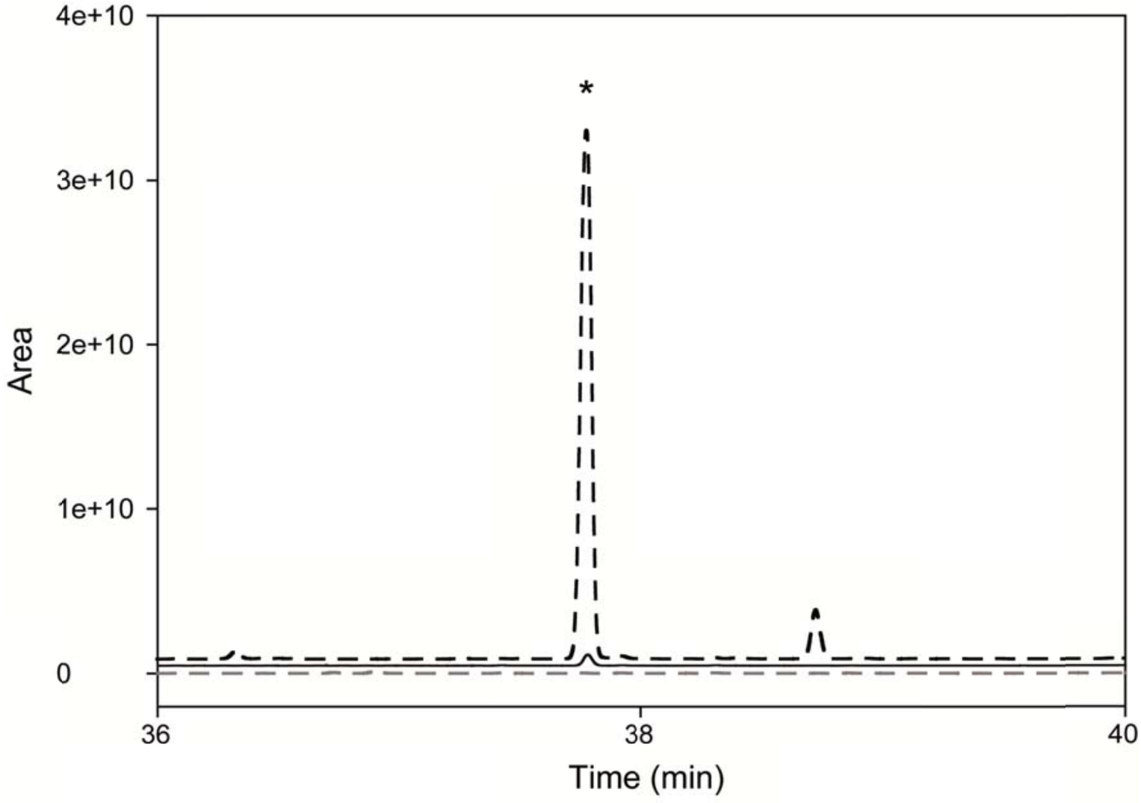

Chromatographic profiles illustrating β-caryophyllene emission of different *Arabidopsis thaliana* lines used in olfatometry assays. WT, wild type plants (black solid line); KO, At5g23960 knock out line (dashed grey line) and OE, At5g23960 overexpression line (dashed black line). *, indicates β-caryophyllene peak.

Suppl. Fig. 4

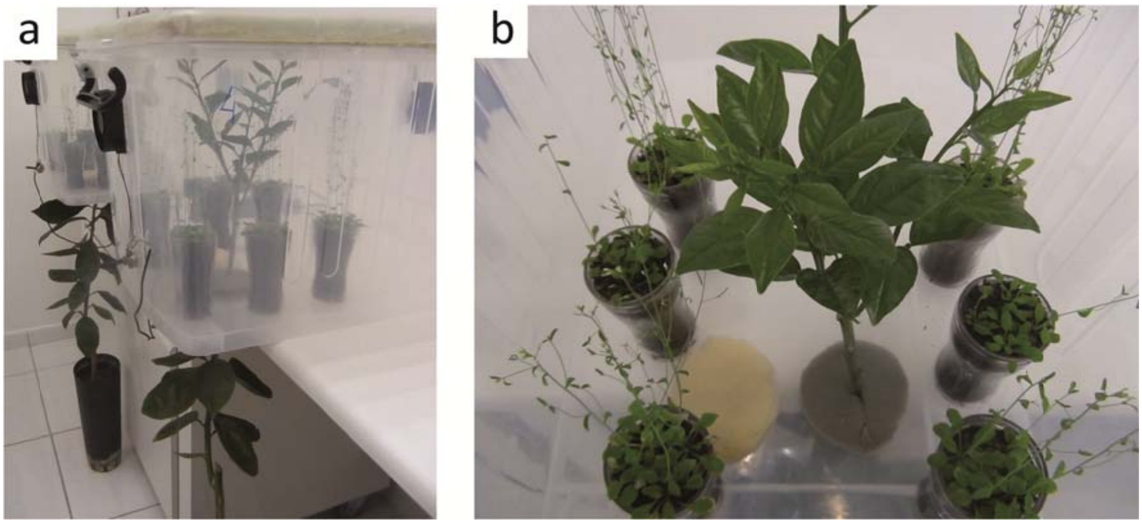

a. Image of an on-going behavioral assay, which shows the way for introducing citrus plant material into the boxes. **b.** Detail of how *Arabidopsis* plants are arranged on both sides of the orange one.

Suppl Fig 5

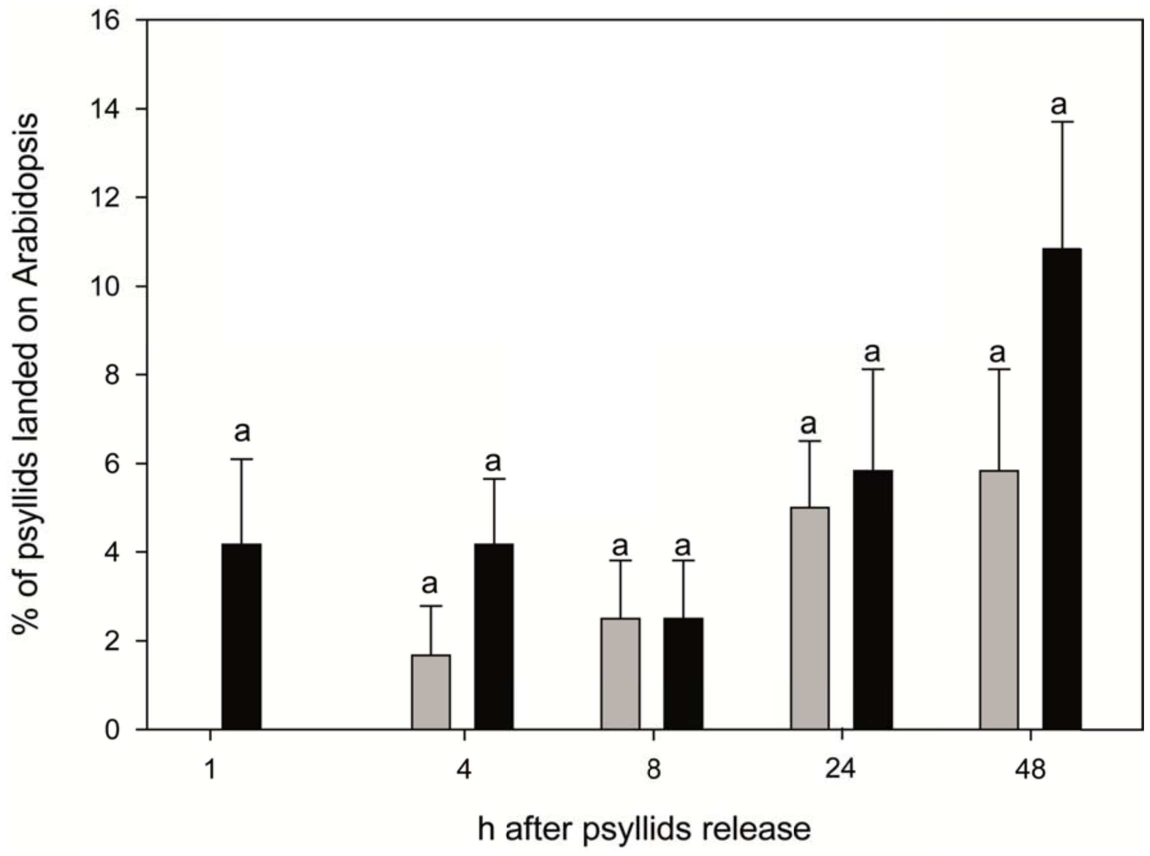

Percentage of *D. citri* psyllids settled 1, 4, 8, 24 and 48 hours after their release on different *Arabidopsis* lines: At5g23960 KO line (grey bars) and At5g23960 OE line (black bars). For a given time, the bars with different letters indicate significant differences between the treatments tested (ns, * P < 0.05). Error bars represent SE (n=12 independent test, 10 psyllids released in each test).

Table S1. Flowering and fructification parameters for WT, At5g23960 knock out (KO) and At5g23960 overexpression (OE) *Arabidopsis* lines.

Line	Open flowers	Closed flowers	Siliques
**WT**	13,8 ± 1,24a	64,6 ± 6,01a	8,2 ± 1,83a
**KO**	14,0 ± 1,30a	64,4 ± 3,37a	12,0 ± 1,87a
**OE**	13,8 ± 0,97a	69,4 ± 5,24a	9,6 ± 2,38a

Data were analysed by paired *t*-test. In each column, same letter indicate non-significant differences between lines ($p < 0.01$).

Supplementary video 1. Smoke test performed to check the correct settlement of the air flow through the device designed to evaluate *Diaphorina citri* response to olfactory and visual cues.
